# Sinus Floor Augmentation with Synthetic Hydroxyapatite (NanoBone^®^) in Combination with Platelet-Rich Fibrin: A Case Series

**DOI:** 10.3390/biomedicines12081661

**Published:** 2024-07-25

**Authors:** Luís Francisco, Manuel Francisco, Rosana Costa, Miguel Nunes Vasques, Marta Relvas, António Rajão, Luís Monteiro, Paulo Rompante, Fernando Guerra, Marco Infante da Câmara

**Affiliations:** 1 Oral Pathology and Rehabilitation Research Unit (UNIPRO), University Institute of Health Sciences (IUCS-CESPU), 4585-116 Gandra, Portugal; a28133@alunos.cespu.pt (L.F.); rosana.costa@iucs.cespu.pt (R.C.); miguel.vasques@iucs.cespu.pt (M.N.V.); marta.relvas@iucs.cespu.pt (M.R.); luis.monteiro@iucs.cespu.pt (L.M.); paulo.rompante@iucs.cespu.pt (P.R.); 2 Clinical Assistant Professor Postgraduate Implant Program, Famalicão Unit, University Institute of Health Sciences (IUCS-CESPU), Av. Marechal Humberto Delgado, 14, 4760-012 Vila Nova de Famalicão, Portugal; mfranciscomc@gmail.com; 3 Department of Medicine and Oral Surgery, University Institute of Health Sciences (IUCS-CESPU), 4585-116 Gandra, Portugal; 4 Associate Laboratory i4HB—Institute for Health and Bioeconomy, University Institute of Health Sciences (IUCS-CESPU), 4585-116 Gandra, Portugal; antonio.rajao@iucs.cespu.pt; 5 Applied Molecular Biosciences Unit (UCIBIO), Translational Toxicology Research Laboratory, University Institute of Health Sciences (1H-TOXRUN, IUCS-CESPU), 4585-116 Gandra, Portugal; 6 Institute of Oral Implantology and Prosthodontics, Faculty of Medicine, University of Coimbra, 3000-075 Coimbra, Portugal; fguerra@ci.uc.pt; 7 Center for Innovation and Research in Oral Sciences (CIROS), Faculty of Medicine, University of Coimbra, 3000-075 Coimbra, Portugal; 8 Laboratory of Hard Tissues, Dentistry Department, Faculty of Medicine, University of Coimbra, 3000-075 Coimbra, Portugal; 9 Coordinator of the Postgraduate Implant Program, Famalicão Unit, University Institute of Health Sciences (IUCS-CESPU), Av. Marechal Humberto Delgado, 14, 4760-012 Vila Nova de Famalicão, Portugal

**Keywords:** platelet-rich fibrin, biomaterials, maxillary sinus augmentation, sinus floor augmentation

## Abstract

Several techniques have been described for maxillary sinus graft augmentation, including the lateral window technique and crestal approach with osteotomes or osseodensification. Platelet-rich fibrin has been used in maxillary sinus lift procedures due to its ability to accelerate soft and hard tissue healing. The aim of this study was to evaluate the potential of PRF in combination with the synthetic hydroxyapatite NanoBone^®^ to enhance bone regeneration in sinus floor elevation with the lateral window technique. Out of the 50 individuals screened in a preoperative assessment visit from the CESPU—Famalicão clinical unit and intervened upon between January 2023 and December 2023, only 6 patients who met the study’s inclusion criteria consented to participate. In a split-mouth study, twelve sinus graft surgeries were carried out. Our observations reveal that for the test group (NanoBone^®^/PRF), there is a 27.5 ± 4.9% increase new vital bone, 23.0 ± 3.7% increase in inert bone particles, and 49.4 ± 2.8% increase in connective tissue. Meanwhile, for the control group (NanoBone^®^), there is a 19.5 ± 3.0% increase in new vital bone, 23.4 ± 5.7% increase in inert bone particles, and 57.0 ± 3.5% increase in connective tissue. The results strongly indicate that mixing liquid PRF with NanoBone^®^ does not have a negative influence on the amount of viable bone formation, and it seems to slightly increase the amount of new bone formation and revascularization in sinus bone graft procedures with the lateral window technique compared to the single use of NanoBone^®^.

## 1. Introduction 

Dental implants are applied in the oral rehabilitation of edentulous posterior maxillae. A prerequisite for implant placement is an adequate bone height and width [1].

The extraction of posterior maxillary teeth can trigger significant bone loss in both vertical and horizontal dimensions, which may preclude implant placement.

Several techniques have been described for maxillary sinus graft augmentation, such as the lateral window technique and crestal approach with osteotomes or osseodensification [2,3,4]. The main difference between the lateral window technique and crestal techniques is the type of bone graft used and the use of an immediate or delayed approach [1].

Knowledge of the anatomy of the maxillary sinus is essential for carrying out this surgical procedure, thus preventing possible complications from arising during sinus elevation.

Numerous grafting materials can be used in sinus graft procedures, such as autogenous bone, demineralized freeze-dried bone allograft (DFDBA), synthetic alloplastic graft, xenograft, and growth factors [5]. Although there is widespread debate on which is the ideal bone graft material, autologous bone is considered the gold standard due to its osteogenic, osteoinduction, and osteoconduction properties [5]. The healing period for maxillary sinus augmentation using autologous bone graft is approximately 6 months, which is the time needed for bone integration and creeping substitution [1]. 

An alternative to autogenous bone grafting is the use of alloplastic, xenograft, or synthetic biomaterials. The bone maturation of these materials takes approximately 9 months [1]. 

Platelet-rich fibrin (PRF) has been used in maxillary sinus lift procedures due to its ability to repair and regenerate bone. PRF is an autologous platelet concentrate containing leukocytes [6]. This procedure, described by Choukroun J, consists of centrifuging the patient’s blood after venipuncture [1]. 

The blood-derived product obtained after the centrifugation of a blood sample is an autologous fibrin matrix that can be mixed with any bone graft substitute or can replace bone graft materials entirely in sinus graft procedures [7,8].

The aim of this clinical–histological study was to evaluate the potential of PRF in combination with NanoBone^®^ Artoss Inc. (St. Cloud, MN, USA) (synthetic hydroxyapatite) to enhance bone regeneration in sinus floor elevation with the lateral window technique.

## 2. Materials and Methods

### 2.1. Study Design

This clinical trial study is reported according to CONSORT guidelines [9]. The interventions were approved by the Ethical Committee of the University Institute of Health Sciences (reference: CE/IUCS/CESPU-18/23) and performed according to the Declaration of Helsinki. The study has been registered in the ISRCTN registry (registration number: ISRCTN99349253). 

### 2.2. Patient Selection

The participants provided their informed consent after being thoroughly enlightened about the goal and methods of the study both orally and in writing.

A meticulous clinical examination, an assessment of oral hygiene, and a detailed analysis of the patients’ medical and dental histories comprised the initial evaluation of each patient. The study’s participants had to be at least eighteen years old and have healed edentulous sites on the posterior maxillae region with a residual bone height of 5 mm or less to facilitate the placement of implants requiring sinus graft procedures. Alcoholism, smoking, drug abuse, diabetes, heart disease, bleeding disorders, weakened immune systems, radiation exposure, past or ongoing use of steroids or bisphosphonates, sinus pathology, and prior bone augmentation were among the exclusion criteria.

Out of 50 individuals screened in a preoperative assessment visit from the CESPU—Famalicão clinical unit and intervened upon between January 2023 and December 2023, only 6 patients who met the study’s inclusion criteria consented to participate. In a split-mouth study, twelve sinus graft surgeries were carried out.

Two experienced examiners (M.I.d.C. and M.F.) carried out a full clinical examination of the mouth and the surgical procedure. 

### 2.3. Preoperative Radiographic Planning

The preoperative radiographic assessment involved the initial screening of patients using cone–beam computed tomography (CBCT, NewTom^®^ Go Ref. 70BE3D, Cefla S.C., Imola, Italy) and a panoramic X-ray. The condition of the Schneiderian membrane, the patency of the ostium, the existence of antral septa and other pathologies that could affect the alveolar bone, the level of pneumatization of the sinus, the thickness of the Schneiderian membrane, and other factors were assessed using CBCT images.

### 2.4. Presurgical Phase

All patients underwent scaling 8 days prior to the implant surgery. During this phase, preoperative instructions were given:

- To eat a light diet, avoiding fatty, fried, laxative, and fermentable foods (milk, cheese, and bananas) on the day of surgery;
- To not to wear jewelry or makeup;
- To not to take medication based on acetylsalicylic acid (aspirin) in the 4 days before surgery;
- To begin using 0.12% chlorhexidine gluconate mouthwash (Bexident^®^ Post Isdin Laboratories, Barcelona, Spain) 48 h prior to surgery (three times a day for two weeks) as well as a tongue scraper;
- To start antibiotic therapy 36 h before surgery (500 mg of levofloxacin) twice daily for 8 days.

### 2.5. PRF Preparation

In order to obtain the PRF before the sinus elevation, blood samples from the patients were obtained in the operating room throughout the procedure. The dried monovettes without anticoagulant were inserted in an Intralock^®^ International, Inc. Centrifuge (Boca Raton, FL, USA) for 3 min at 2700 rpm following the blood draw to obtain liquid fibrin.

NanoBone^®^ particles were agglomerated in a sterile container with the liquid fibrin and applied to the surgical site in order to achieve the sinus augmentation.

A Straumann^®^ Fex collagen membrane was applied in the lateral window access point after the full packing of the maxillary sinus.

### 2.6. Surgical Procedure

The procedure was carried out under local anesthetic using Articaina Inibsa^®^. A crestal incision in keratinized gingiva as well as a posterior releasing incision were used to access the lateral maxillary sinus wall. A piezoelectric insert (Acteon Satelec^®^ SL2) was used to outline a bone window measuring roughly 15–20 mm and was continuously irrigated with saline solution at a rate of 60 mL/min. Subsequently, the Schneiderian membrane was lifted utilizing the piezoelectric inserts (Acteon Satelec^®^ SL4 and SL5), which were continuously irrigated with saline solution at a rate of 60 mL/min. To enhance the sinus floor augmentation, 1.2 mL NanoBone^®^ with a particle size of 1.0 mm was introduced into the sinus cavity after the Schneiderian membrane was carefully elevated without being perforated. 

NanoBone^®^ alone hydrated with sterile saline was used to fill the sinus cavity in 6 sinuses (control group). Liquid PRF was added to the bone graft particles (test group) in the 6 contralateral sinuses.

**Figure 1:**
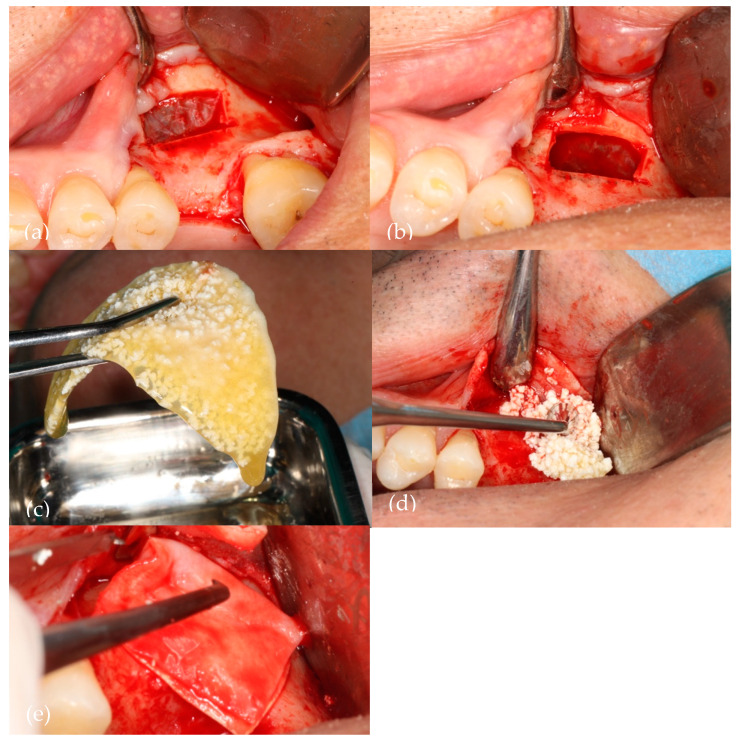
A visual representation of the surgery. (**a**) Bony window with Acteon Satelec^®^ piezoelectric device; (**b**) Schneiderian membrane elevated; (**c**) Aggregation of NanoBone^®^ with liquid fibrin; (**d**) Biomaterial insertion in the sinus cavity; (**e**) Straumann^®^ Fex collagen membrane over the bony window.

Figure 1 illustrates the sinus lift procedure (liquid PRF/NanoBone^®^).

### 2.7. Postoperative Instructions

The following postoperative instructions were given to avoid increased edema (swelling), pain, bleeding, and infections:

- Avoid anything that creates pressure in the nasal cavity.
- For the four weeks after sinus graft surgery, do not sneeze or nose blow while holding the nose. If specified, this duration might be extended.
- Be careful to sneeze with your mouth open if necessary.
- Avoid using straws for drinking and avoid spitting.
- Avoid bearing down, which includes lifting heavy objects, blowing up balloons, playing musical instruments that require a blowing motion, and engaging in any other activity that raises nasal or oral pressure. Avoid scuba diving and flying in pressurized aircraft (as these activities will increase sinus pressure).

After surgery, bleeding usually ends a few hours later. For the first three days, some leaking or sporadic bleeding is typical.

- For thirty minutes, leave the gauze pad(s) immediately on the surgery site(s).
- Avoid biting the pad. Until the bleeding stops, apply hard biting pressure for 30 min and swap out the pad every 30 min.
- After attempting the treatments mentioned above, if bleeding persists, moisten a black tea bag, place it over the surgical site(s), cover it with gauze pads, and bite firmly for at least half an hour.
- Steer clear of demanding activities for a week. After surgery, intense physical activity may result in throbbing and bleeding.

Swelling is common after most oral surgeries. Usually, the swelling worsens for three days before starting to improve on the fifth day. For the first 36 h, you should apply cold compresses to your face for 20 min and 5 min to help minimize swelling and pain. If sitting or sleeping, elevate the head with two or three pillows or in a reclining chair. Switch to low heat after 36 h to help reduce swelling.

- After surgery, bruising may occur based on the procedure and the patient’s propensity for bruising. Like any other bruise, bruises usually disappear within a few days to two weeks.

Regarding the oral hygiene procedure, you should start cleaning your teeth again, but more softly. If using toothpaste hurts, try brushing your teeth without it.

Restraining yourself from rinsing or spitting could cause the blood clot to come loose and cause discomfort or bleeding. The 0.12% chlorhexidine gluconate mouthwash should be continued to reduce plaque formation. 

Alcohol should not be consumed for at least seven days after surgery. In addition to delaying wound healing, alcohol consumption is one of the main causes of infections.

Partial prostheses, including flippers, should not be used immediately after surgery until your post-operative appointment, unless there are specific instructions to the contrary. When it is placed, it must not touch the gums in the surgery area. Pressure from the partial denture can lead to bone graft loss.

A lot of fluids should be consumed following surgery, preferably water. Drinks should not be sucked through a straw. Skip any carbonated beverages for a full 72 h. As soon as it feels comfortable (typically after seven days), go back to your regular diet, starting with softer foods.

Regarding medication, antibiotic therapy should be continued, and 1 g of paracetamol should also be taken 3 times a day for pain control management. The use of a Mometasone spray is also advised (1 application in each nostril twice daily for 3 days) due to the reduction in the activity of the cilia of the Schneiderian membrane and the thickening of mucous secretion.

After the post-operative indications were completed, the patients were scheduled for suture removal ten days after surgery.

### 2.8. Harvesting of the Bone Specimen

Dental implants can usually be inserted after the grafted bone has effectively merged, usually within 6 to 9 months. The dentist’s assessment and each patient’s unique recovery rate may influence the precise timing. In this study, implants were placed six months after sinus floor augmentation in both groups. Using a 3 mm diameter trephine bur, a bone biopsy from the augmented site was obtained from both the control group and test group during this treatment. The trephine drill was centered in the previous sinus lateral bone window. The bone samples were then stored in a sterile vial with 10% formaldehyde.

**Figure 2:**
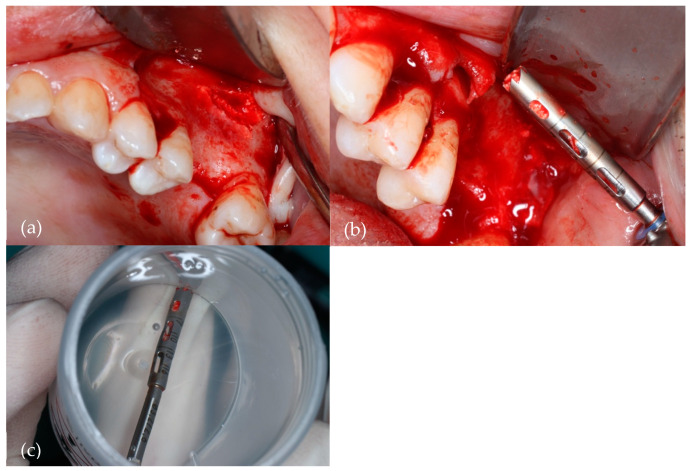
Harvesting of the bone specimen with a 2.5 mm diameter trephine bur. (**a**) Surgical site; (**b**) Trephine drill with collected bone; (**c**) Trephine drill with bone specimen in a 10% formaldehyde sterile vial.

Figure 2 illustrates the collection of the bone specimen. 

### 2.9. Sample Processing and Analysis

An analysis, both qualitative and quantitative, was conducted on the processed study material.

The hard tissue was carefully dissected before the material was extracted en bloc. Using high-precision Exakt^®^ equipment (Exakt^®^ Technologies, Oklahoma City, OK, USA), the collected samples were processed using an undecalcified approach that produced high-quality histological pictures without morphological distortions of relevant structures or meaningful artifacts. In terms of staining techniques, toluidine blue was employed.

Histological processing was carried out following the protocol for non-decalcified techniques, as recommended and described by Donath K [10]: the preparation of histologic sections using the cutting–grinding technique for hard tissue and other material not suitable for sectioning using routine methods: Equipment and Methodical Performance (Exakt^®^—Kulzer—Publication, Norderstedt.), without any attempt to remove the harvested tissue from inside the specimen. This preservation made it possible to maintain the proper orientation of the slides, making it possible to see all the tissue from lateral to medial.

Quantitative analysis was carried out by capturing images from the aforementioned video camera (Nikon^®^ SMZ 1500, Tokyo, Japan) coupled with a stereomicroscope (Optronics DEI 750D CE, Goleta, CA, USA) and connected to a PC computer (Intel^®^, Pentium^®^ V). 

Histomorphometry was carried out using image analysis Bioquant^®^ Nova (Bioquant—Image Analysis Corporation, Nashville, TN, USA) software. This program is able to distinguish the different dye affinities of the tissues and components of the sample, converting this information into the quantification of areas, three-dimensional reproductions, determination of densities, and other more specialized parameters. This evaluation system allows for greater objectivity and precision compared to other evaluation systems, such as radiomorphometry or systems with degree scales.

The images were assessed at a magnification of 10 × 0.5 for qualitative analysis. 

Calibration of the program and analysis were always carried out by the same operator. All sessions were preceded by an intra-examiner calibration check.

Histomorphometry involved the use of the total area of the bone tissue sample as a reference, which was taken as the standard area for all defects in order to minimize measurement bias. 

The following parameters were assessed in the histomorphometric analysis:

(a) Quantification of the percentage of particles:
  - Area occupied by particles/defect area × 100%
(b) Quantification of the percentage of new bone tissue:
  - Percentage of new bone tissue = (area of new bone tissue/area of defect) × 100%
(c) Quantification of connective tissue:
  - Connective tissue = (area of connective tissue/area of defect) × 100%

### 2.10. Statistical Analysis

The resulting data of the bone specimens were presented as the mean value along with the standard deviation. To assess statistical significance, an analysis was performed using GraphPad Prism (version 9.5.0, United States).

A two-way analysis of variance (ANOVA) was performed using Tukey’s multiple comparisons post hoc test to identify significant differences between the means, with a significance level set at *p* < 0.001.

## 3. Results

Out of 50 individuals screened in a preoperative assessment visit from the CESPU—Famalicão clinical unit, only 6 patients who met the study’s inclusion criteria consented to participate.

**Figure 3:**
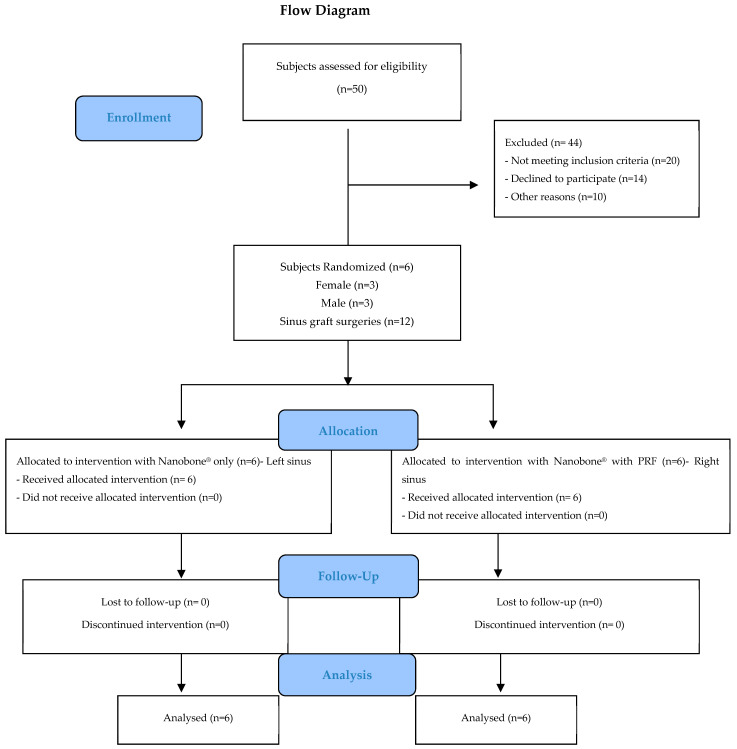
CONSORT flowchart.

Figure 3 illustrates the design of the study in the form of a CONSORT diagram.

After bone sample collection, histomorphometric analysis was carried out on the samples from both the control group (NanoBone^®^ alone) and test group (NanoBone^®^/liquid fibrin). 

Under light microscopy and upon extensive evaluation of the histological lamellas of both groups, there seem to be differences between the two sets of results under analysis. 

**Figure 4:**
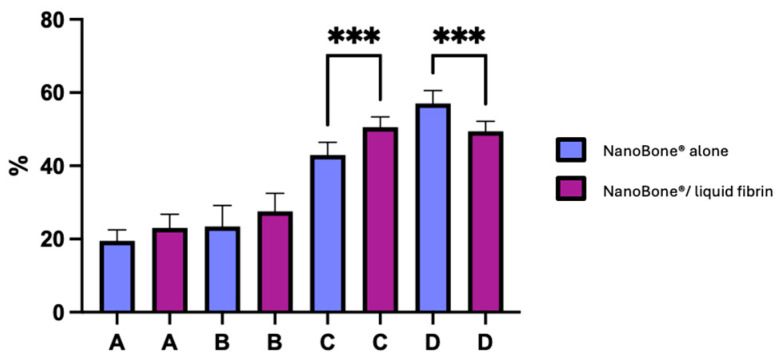
Newly formed bone, residual bone graft material, and connective tissue for both control (NanoBone^®^ alone) and test groups (NanoBone^®^/liquid fibrin). Bars represent the mean and standard deviation of individuals results. (A) New vital bone, (B) particles, (C) new vital bone + particles, and (D) connective tissue. (*** *p* < 0.0005).

Figure 4 illustrates the percentage of new bone formation in both groups.

The rate at which vital and inert bone is present in the bone trabecular sections allows one to assess the significance of turnover. Our observations reveal that for the test group (NanoBone^®^/PRF), there is a 27.5 ± 4.9% increase in inert bone particles, 23.0 ± 3.7% increase in new vital bone, 50.5 ± 2.8% increase in new vital bone + particles, and 49.4 ± 2.8% increase in connective tissue. Meanwhile, for the control group (NanoBone^®^), there is a 19.5 ± 3.0% increase in new vital bone, 23.4 ± 5.7% increase in inert bone particles, 43.0 ± 3.5% increase in new vital bone + particles, and 57.0 ± 3.5% increase in connective tissue. Osteoid tissue’s significance in both group samples provides proof of significant turnover. After six months of bone healing, the histomorphometric results of the test group (NanoBone^®^/PRF) seem marginally better than those of the control group (NanoBone^®^). Statistically significant differences were found in two parameters: between the test group and the control group with regard to new vital bone + particles and between the control group and the test group with regard to connective tissue (*p* < 0.0005 for both parameters).

The histological characteristics of the images under study were labeled from A to G, as follows: **A**—osteocytes; **B**—osteoclasts; **C**—amorphous material (NanoBone^®^); **D**—remodelling surface (osteoblasts); **E**—lamellar bone; **F**—Havers system; **G**—immature bone tissue; **H**—connective tissue; **I**—endosteum; **J**—osteoid; **L**—NanoBone^®^ particles.

**Figure 5:**
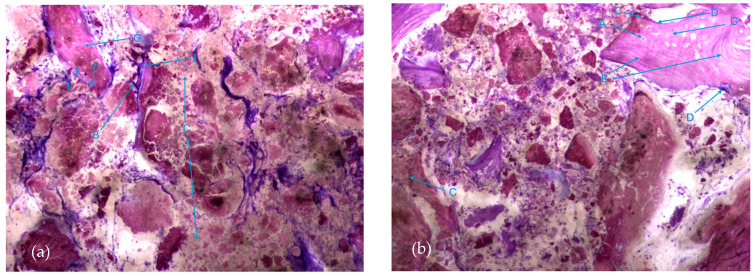
Histologic results in both groups: (**a**) test group; (**b**) control group. 10 × 0.5 magnification and toluidine blue staining.

The most characteristic aspect of the histological images in the test and control groups is the presence of a high density of particles of various sizes and shapes, many of them in a clear and intense process of disintegration/fragmentation (Figure 5a,b).

**Figure 6:**
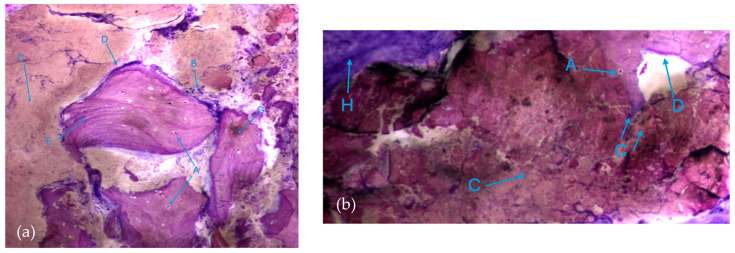
Histologic results in both groups: (**a**) test group; (**b**) control group. 10 × 0.5 magnification and toluidine blue staining.

The results, both in the test group and control group, also exhibited extensive areas occupied by a homogeneous/amorphous material (Figure 6a,b), which, considering its color characteristics, seems to originate from the progressive dissolution of the particles under study. These aggregates generally contain multiple particles with very small dimensions.

**Figure 7:**
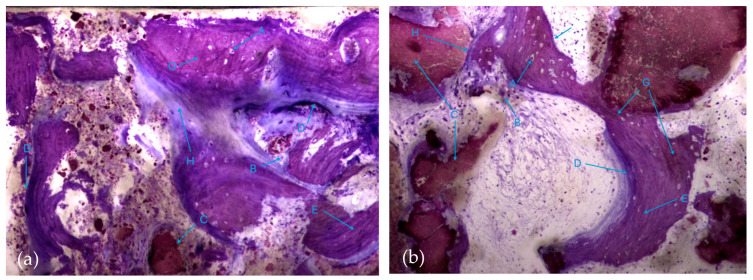
Histologic results in both groups: (**a**) test group; (**b**) control group. 10 × 0.5 magnification and toluidine blue staining.

It is possible to observe, in both groups, the presence of numerous bone trabeculae formed by immature bone tissue containing numerous irregularly arranged osteocytes. It is worth noting the existence of many areas of osteoid tissue, reflecting an active process of osteogenesis (Figure 7a,b).

**Figure 8:**
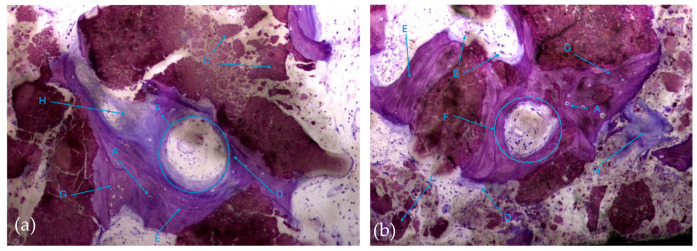
Histologic results in both groups: (**a**) test group; (**b**) control group. 10 × 0.5 magnification and toluidine blue staining.

In this respect, recently formed bone tissue is often seen in close apposition to the particles, although it is not common to find particles completely surrounded by bone tissue. It is interesting to note the displaying continuity between the bone tissue and the particles, with no border/interface between them. It should also be noted that even the particles where bone tissue is directly attached are themselves in the process of disintegration in both the control group and test group (Figure 8a,b). 

**Figure 9:**
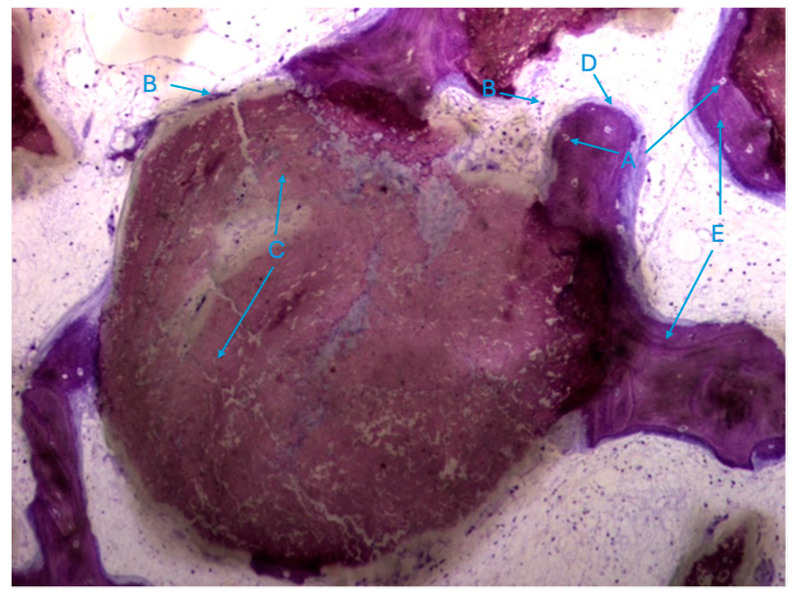
Histologic results in the test group. 10 × 0.5 magnification and toluidine blue staining.

It was also possible to identify numerous osteoclasts, both on the surface of areas of immature bone tissue and on the surface of the particles (Figure 9), demonstrating the presence of active resorption phenomena. 

**Figure 10:**
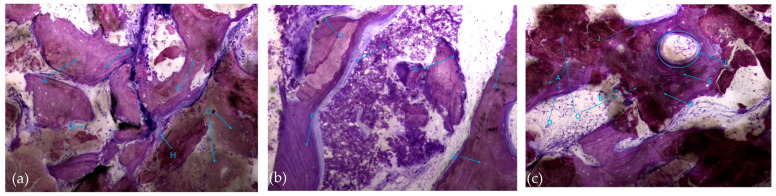
Histologic results in both groups: (**a**,**b**) test group; (**c**) control group. 10 × 0.5 magnification and toluidine blue staining.

Also noteworthy is the observation of areas of lamellar bone tissue formed by thick trabeculae in the test group (Figure 10a), showing the existence of Havers systems that are already completely formed (Figure 10b) or in the formation process in both groups (Figure 10c).

**Figure 11:**
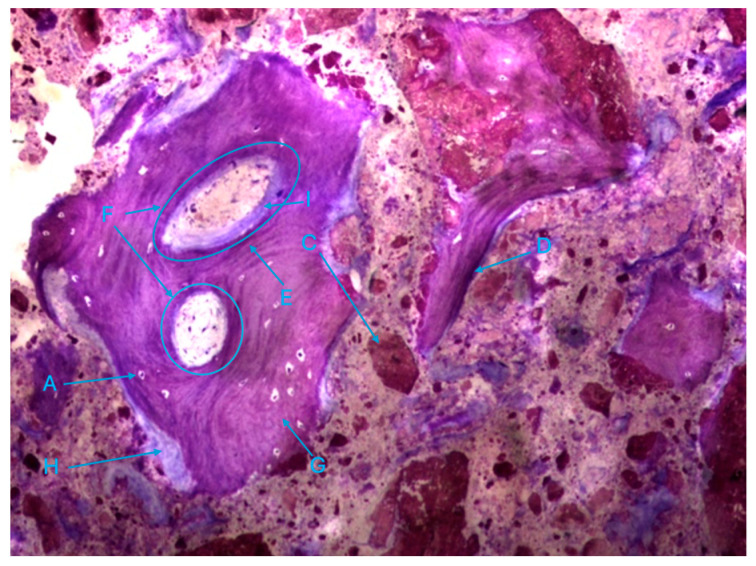
Histologic results in the control group. 10 × 0.5 magnification and toluidine blue staining.

The trabeculae show signs of integrated particles, which is characteristic of these trabeculae (Figure 11).

### 3.1. General Considerations in the Treatment of Bone Sample Specimens

In order to visualize, in detail, the internal tissues of the bone specimen, longitudinal rectangular sections were taken. 

The longitudinal rectangular sections taken from the biopsy allowed for the correct visualization of the entire length of the tissue.

The histological images showed that the method used to prepare the material was successful, resulting in well-preserved sections of good quality. Toluidine blue was used to select the staining methods.

In order to provide a better understanding of the description of the results, we will sometimes use a division of the histological material into thirds, respecting the length, from lateral to medial. We will begin by describing the slides obtained from maxillary sinuses filled only with bone substitute material (NanoBone^®^) hydrated with saline, of synthetic origin, in 1 to 2 mm granules, and then those in which this biomaterial was mixed with liquid fibrin.

### 3.2. Maxillary Sinuses Filled with Hydrated Bone Substitute Material or Mixed with Liquid Fibrin

In the longitudinal sections, it is possible to appreciate the biomaterial granules presented along the entire length of the section. There are also areas of cancellous bone tissue made up of bone trabeculae, areas of lamellar bone, and others, possibly in smaller quantities, of immature bone in which the characteristic irregularly arranged osteocytes are visible. Bone tissue formation activity at the site where the maxillary sinus floor elevation intervention was carried out was evident, as evidenced by the presence of bone tissue in contact with some of the biomaterial granules along the entire length of the cut, apparently in a greater quantity near the lateral third and, above all, at its medial limit. In fact, the trabeculae were more sparse in the middle third. However, it should be noted that there may be some individual physiological variabilities in the anatomical design of the sinus. It was also possible to see the existence of bone tissue between the surroundings and between some particles forming bridges between them, demonstrating the osteoconductive effect of the biomaterial’s inorganic mineral granules.

The ossification processes found are of intramembranous origin.

In some histologic images, clot remnants are visible and are more prevalent in the lateral and medial thirds, certainly related to the collection method and consequent rupture of blood vessels.

The entire length of the section also shows many granules surrounded only by loose connective tissue, with no signs of bone formation activity. The implanted material did not reveal any type of histological image compatible with a local adverse reaction, and no foreign body giant cells or other inflammatory cells were detected.

Where it was identified that bone formation activity had occurred, the formation of bone trabeculae was visible, with organized lamellar bone tissue, bone apposition on the particles, and the formation of bone tissue bridges between them surrounded by medullary spaces filled with loose connective tissue, with fibroblast-like cellular elements and blood vessels.

On the other hand, some aspects related to the degradation of the biomaterial granules can be observed, namely what appears to be their disintegration into smaller particles, the difficulty in defining the limits of granules, and the presence of osteoclastic activity on its surface. When there is bone tissue-forming activity on the surface of the granules, most of the time, it only occurs on part of the surface, and it is very rare to find granules completely surrounded by bone apposition.

## 4. Discussion

The purpose of this clinical trial was to evaluate the potential of PRF in combination with synthetic hydroxyapatite NanoBone^®^ to enhance bone regeneration in sinus floor elevation with the lateral window technique.

Due to pneumatization of the maxillary sinus and atrophy of the alveolar bone ridge, the edentulous posterior maxilla often provides a limited bone volume [11]. The remaining bone is often of type IV quality, which makes implant rehabilitation more difficult in this region [11].

Tooth loss is often followed by a complex biophysical process known as residual ridge resorption. This process reaches its highest point in the first year after tooth loss, and then, resorption continues at a slower but steady pace in the following years [12,13]. All edentulous patients suffer from bone resorption, which is a chronic, gradual, and irreversible process [14].

In 1988, to simplify the description of the residual ridge and thus aid communication between clinicians, Cawood et al. [15] developed a classification of edentulous jaws based on a randomized cross-sectional study. According to this classification, the residual ridge is classified into six classes (Class I to Class VI) according to the type of bone loss in both the height and width [15]. In the posterior maxillary region, bone loss is both vertical and horizontal (from the buccal aspect). 

The pneumatization of the sinus combined with alveolar bone resorption leads to an insufficient bone quantity for implant placement [16,17]. 

For this reason, sinus mucosa is required to be lifted from the sinus floor (sinus lift augmentation), and new bone formation is achieved by using bone substitute materials that are accommodated below the Schneiderian membrane [16,17].

A complete and ideal graft material has not yet been found; autogenous bone possesses several advantages such as osteogenic, osteoinductive, and osteoconductive properties, but it presents some difficulty in obtaining autogenous bone graft in sufficient quantities and requires some more complex expertise from the surgeon to address donor areas [18].

In terms of new bone formation, the use of autogenous bone in maxillary sinus augmentation is predictable and successful; however, donor site morbidity is inevitable [19]. The osteoconductive properties of biomaterials and allogeneic sources have enabled them to be widely used in maxillary sinus augmentation procedures to replace autogenous bone, which harbors osteogenic cells that induce direct bone regeneration [20]. With the advent of blood matrix technology, biomaterials extracted from the patient’s blood, such as platelet-rich fibrin (PRF), have recently been more frequently reported in sinus floor augmentation treatments. PRF has been used in dental practices and is simple to handle and prepare [20]. This natural and optimized blood clot could be used during a sinus lift for the protection of the sinus membrane or to improve bone graft maturation. PRF promotes the growth and differentiation of osteoblasts, among many other types of bone cells [21].

In the last few years, PRF has been used alone or combined with different grafting materials [22]. Evidence has shown that the use of PRF alone or its association with various grafting materials in maxillary sinus floor augmentation demonstrated successful bone regeneration [23,24]. According to our results, the use of PRF mixed with synthetic hydroxyapatite (test group) exhibited increased vital bone formation in comparison with synthetic hydroxyapatite (control group): 23.0% versus 19.5%, respectively. This can be explained by two positive factors: the osteoconductive capacity of NanoBone^®^ and the positive influence of liquid fibrin on enhancing bone cell differentiation. 

A study by Ortega-Mejia et al. [7] showed a slightly higher percentage of new bone formation in the PRF group in comparison to the use of grafting biomaterials alone. These results could be explained by its osteoinductive qualities and better revascularization process, accelerating the healing period of the bone tissue [25]. 

A study by Tanaka et al. [26] also showed a higher percentage of new bone formation in their histological evaluation of sinus lift using deproteinized bovine mixed with PRF. 

In their study, Choukroun et al. [1] revealed that after 4 and 8 months of healing, respectively, there were no changes in the newly created bone between the PRF mixed with freeze-dried bone allograft (DFDBA) and the DFDBA alone. This suggests that the addition of PRF could shorten the healing period prior to implant placement.

A study by Zhang et al. [27] showed that PRF combined with Bio-Oss^®^ had no significant synergistic effect on new bone formation or the graft volume. These different results could be due to the different bioactive properties of Bio-Oss^®^ and NanoBone^®^. This study’s findings are consistent with the study by Choukroun et al. [1] in that poor bone growth is primarily located farther from the sinus floor. They suggest that, as the source of precursor cells, the sinus floor is crucial to bone repair. Precursor cell migration to the site may be less stimulated by PRF mixed with Bio-Oss^®^ than by PRF combined with FDBA [27]. In our study, we harvested biopsies from the site furthest from the sinus floor; even so, there was an increase in cell activity in the NanoBone^®^ and liquid PRF group. The results shown in the study by Zhang et al. [27] could be explained by the lack of precursor cells in the PRF group combined with Bio-Oss^®^. Furthermore, according to several studies, the slow resorption property of this bone substitute slows the replacement of new bone formation [28,29]. 

Liu et al. [30] carried out a study to compare the in vitro biocompatibility of Bio-Oss^®^ versus NanoBone^®^ and their ability to support and promote the proliferation of human osteoblasts. According to their study, both materials showed low cytotoxicity and excellent biocompatibility. However, the test for cell proliferation was superior for NanoBone^®^, which may explain the difference between our results and those found in the study by Zhang et al. [27]. 

During bone remodeling, osteoclasts’ degradation of biomaterials is a crucial and desired process. It has been shown that osteoclasts’ biodegradation rate of NanoBone^®^ appears to have more similarity to the body’s natural process of remodeling bone [31]. Targeted vascularization facilitates the migration of osteoblasts to vascularized sites and can accelerate the creation of new bone [31]. Prior studies showed that when placed in vascularized connective tissue, several synthetic bone graft materials, such as NanoBone^®^ replacements, were covered and degraded by osteoclasts, resulting in resorption lacunae and resorptive trails [31,32]. Our results are in accordance with this; histologically, we observed numerous osteoclasts both on the surface of areas of immature bone tissue and on the surface of the particles. 

The fact that NanoBone^®^ is made of nanocrystalline hydroxyapatite, which resembles the crystalline phase of native bone, may be the reason for this material’s biocompatibility. In addition to these characteristics, when PRF is added to this biomaterial, the fibrin clot acts as a biological binder between the various components of the graft and a matrix that allows for the migration of osteoprogenitor cells to the center of the graft, the retention of stem cells, and neo-angiogenesis when mixed with the graft material. This can be explained by the fact that PRF promotes an increase in bone mass and greater revascularization of the produced bone.

A significant limitation of the present study is the small sample size. To truly understand the extent of healing time shortening achieved using PRF, it would be interesting to harvest bone specimens three different times after sinus augmentation (after 4, 6, and 9 months). 

## 5. Conclusions

According to this study, the nanoporous hydroxyapatite used to raise the sinus floor in humans is osteoconductive and promotes the production of new bone in a similar manner to that of most other bone substitute materials. 

The results strongly indicate that mixing liquid PRF with NanoBone^®^ seems to have a positive influence on the amount of viable bone formation; it seems to increase the amount of new bone formation and revascularization in sinus bone graft procedures with the lateral window technique compared to the single use of NanoBone^®^.

In the histological sections, it was possible to see areas of bone remodeling (osteoblasts) and areas of defragmentation of the NanoBone^®^ particles, as well as Havers systems that are indicative of bone formation with some degree of maturity, which shows the osteoconduction capacity of the bone substitute material.

PRF seems to be a reliable method for incorporating bone substitute material and seems to increase the amount of viable bone formation when mixed with the nanoporous hydroxyapatite.

Nevertheless, to validate the current findings and assess the long-term effectiveness of PRF mixed with nanoporous hydroxyapatite, prospective investigations involving larger patient groups and a longer follow-up period are required.

## Data Availability

The data can be accessed by contacting the corresponding author due to ethical reasons.
